# Malaria outbreaks in China (1990–2013): a systematic review

**DOI:** 10.1186/1475-2875-13-269

**Published:** 2014-07-10

**Authors:** Guangyu Lu, Shuisen Zhou, Olaf Horstick, Xu Wang, Yali Liu, Olaf Müller

**Affiliations:** 1 Institute of Public Health, Medical School, Ruprecht-Karls-University, INF 324, 69120 Heidelberg, Germany; 2 National Institute of Parasitic Diseases, Chinese Centre for Disease Control and Prevention (CDC), Shanghai, China; 3 Key Laboratory of Parasite and Vector Biology, MOH, Shanghai, China; 4 WHO Collaborating Centre for Malaria, Schistosomiasis and Filariasis, Shanghai, China; 5 Institute of Global Health University College, University College London, London, UK; 6 Evidence-Based Medicine Centre, School of Basic Medical Sciences, Lanzhou University, Lanzhou, China; 7 Key Laboratory of Clinical Translational Research and Evidence-Based Medicine of Gansu Province, Lanzhou, China

**Keywords:** Malaria, Outbreak, China, Systematic review

## Abstract

**Background:**

China has already achieved remarkable accomplishments in shrinking the malaria burden since the mid-20th Century. The country now plans to eliminate malaria by the year 2020. Looking at the dynamics of malaria outbreaks during the last decades might provide important information regarding the potential challenges of such an elimination strategy and might help to avoid mistakes of the past.

**Methods:**

A systematic review of the published literature (English and Chinese) was conducted to identify malaria outbreaks during the period 1990 until 2013 in China. The main causes of outbreaks as described in these papers were categorized according to whether they were related to population migration, environmental factors, vector and host related factors, and operational problems of the health services.

**Results:**

The review identified 36 malaria outbreaks over the 23-year study period, on which sufficient information was available. They mainly occurred in southern and central China involving 12 provinces/autonomous regions. More than half of all outbreaks (21/36, 58%) were attributed at least in part to population migration, with malaria importation to non- or low-endemic areas from high-endemic Chinese areas (13/15) or endemic countries (2/15) having been the most frequent reason (15/21, 71%). Other main causes were problems of the health services (15/36, 42%), in particular poor malaria case management (10/15, 67%), environmental factors (7/36, 19%), and vector and host related factors (5/36, 14%).

**Conclusions:**

Beside a number of other challenges, addressing population movement causing malaria appears to be of particular importance to the national malaria programme. Strengthening of surveillance for malaria and early radical treatment of cases should thus be considered among the most important tools for preventing malaria outbreaks and for the final goal of malaria elimination in China.

## Background

Malaria was highly endemic in China (estimated 30 million annual cases) before the foundation of the People’s Republic in 1949 [1]. Tremendous efforts have been put into reducing the malaria burden since the launch of the National Malaria Control Programme in 1955. Despite two major outbreaks in the 1960s and 1970s, respectively, the country showed a steady decrease of its malaria burden [2]. By the end of 1990, there were 117,000 malaria cases reported from China [2]. Over the last two decades, China has further reduced the malaria burden, now with a goal of elimination by 2020 [3,4]. However, sporadic outbreaks have consistently been reported during the last two decades [5-7].

*Plasmodium falciparum* and *Plasmodium vivax* are the two main malaria species in China. Falciparum malaria was already restricted to only two provinces (Yunnan, Hainan) by 1998, and vivax malaria accounted for 95% of the indigenous malaria cases in 2012 [8,9]. *Plasmodium vivax* is less responsive to control interventions and much more difficult to eliminate than *P. falciparum*[10]. On the other side, imported falciparum malaria is increasingly seen in many provinces [8,11].

Malaria outbreaks are a clear threat to maintain achievements and to the final goal of elimination in China [12]. They can have various reasons, ranging from environmental causes over parasite/vector characteristics to health system factors and human behaviour aspects [13]. Increasing development in China, which is associated with increased mobility, led to malaria outbreaks in areas where local malaria transmission had previously been interrupted. A strengthened surveillance system contributed largely to active investigation and better reporting of these outbreaks. Therefore, a thorough analysis of the specific reasons for these outbreaks will be helpful to improve the effectiveness of the national malaria programme.

A systematic literature review on causes of published malaria outbreaks in China from 1990 until 2013 has thus been conducted.

## Methods

### Search strategy

All published malaria outbreaks within Mainland China between 1 January, 1990 and 12 September, 2013 were systematically retrieved. English articles were searched from Medline, Web of Science, Embase, the Cochrane Central Register of Controlled Trials and Evidence for Policy and Practice Information and coordinating Centre (EPPI-Centre) by using the search terms ‘malaria’, ‘*Plasmodium vivax*’, ‘*Plasmodium falciparum*’, ‘outbreak’, ‘resurgence’, ‘re-emergence’ ‘relapse’ and ‘China’, ‘People’s Republic of China’. Articles published in Chinese were identified through China National Knowledge Infrastructure (CNKI), China Biology Medicine (CBM), VIP, WanFang database and the website of the Chinese Centre for Disease Control and Prevention by using the same keywords.

### Selection criteria

#### Time period

The study concentrated on the years 1990 until 2013, as data are more complete for this time period and as the reasons for outbreaks from this period are most applicable to the present malaria status in China.

#### Outbreak causes

Only studies that discussed potential main causes for malaria outbreaks were included, irrespective of an implementation of active epidemiological investigations or not.

#### Outbreak definition

Before 2006, there was no malaria outbreak definition in Chinese guidelines, thus and according to WHO, a malaria outbreak was considered as ‘the occurrence of cases of disease in excess of what would normally be expected in a defined area or season’ in the analysis. Since 2006 and according to new Chinese guidelines, malaria outbreaks were more specifically defined as ‘an unusual increase or a new occurrence of autochthonous (indigenous or introduced) cases in a certain area’ [14,15]. In areas of endemic malaria, increase refers to an at least doubling of incidence between two years (starting from at least ten cases per month at village level); in addition at least 3% of the at-risk population has to contract malaria one or more times in stable endemic areas and at least 1% in unstable endemic areas. Regarding areas where malaria has already been eliminated (no local transmission for at least three years), an outbreak is defined as at least five indigenous (mosquito-borne malaria from local infections) cases per month or at least one newly introduced (mosquito-borne malaria from an imported infection) falciparum malaria case at village level. In areas where no local malaria transmission has been observed for at least five years, any newly introduced case is defined as an outbreak. The occurrence of only imported cases not having caused secondary cases is not considered an outbreak.

### Data extraction and analysis

All papers found were carefully read and discussed by two authors (GYL, YLL) and those fulfilling the selection criteria were included in the analysis. The characteristics of malaria outbreaks (e g, population, time period, parasite and vector species, malaria epidemiology, and data collection method) were systematically extracted. Outbreaks were listed geographically according to the following four categories from the Action Plan of China Malaria Elimination (2010–2020) [16].

Analytical categories were identified inductively based on the main suggested outbreak causes in every paper. Two authors (GYL, XW) extracted the main causes independently from each paper, and then two authors (GYL, OM) classified them into analytical categories. The content of each paper was systematically checked for all causes relevant to each category. Finally, all causes identified were classified into four main non-overlapping categories: (1) population migration (e g, person migration from low- to high-transmission areas or *vice versa*); (2) environmental factors (e g, climatic changes, man-made breeding sites and natural disaster); (3) vector and host related factors (e g, increased human-vector contact or vector capacity); and, (4) operational problems of the health services (e g, vector control ceased, problems with case management and weakening of surveillance).

### Quality appraisal

As there is no recognized technical tool for quality appraisal of field reports in non-interventional systematic reviews, a criteria for judgement of the credibility and consistency of included reports was developed. In this analysis, credibility refers to whether the suggested main causes of malaria outbreaks originated only from assertion or speculation without provision of quantitative data from detailed investigation or from evidence-based conclusion based on active quantitative or qualitative epidemiological investigation. Consistency of included outbreaks was furthermore crosschecked if outbreaks and their characteristics were reported in more than one publication.

In case of disagreement during paper selection for inclusion, data extraction, analytical category identification and quality appraisal processes, cases were discussed until a consensus had been reached.

## Results

**Figure 1:**
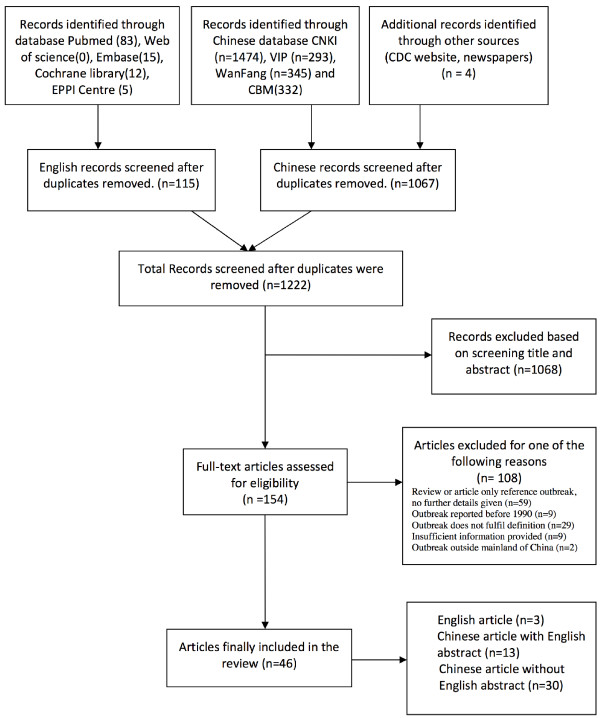
Systematic selection process of malaria outbreak articles in China into the review.

The initial search yielded 1,564 records, of which 1,222 remained after removing duplicates (Figure 1). Among them, 154 articles appeared to describe or allude to malaria outbreaks so the full texts were assessed for eligibility. Of these 154 articles, 59 were excluded because the information in the paper was repeated in more detail in other papers; 29/154 articles describing 17 epidemic events were excluded because they do not meet the outbreak definition. For example, the majority (13/17) of the reported epidemics were based on imported malaria cases with no secondary cases. A few of these epidemics were small family clusters (2/17) or caused by parasite transmission through blood donations (2/17). Nine of the 154 articles were excluded because of insufficient information provided; nine were excluded because the reported events occurred before 1990; two articles were excluded because the reported events occurred outside Mainland China. Thus, a total of 46 articles describing 36 outbreaks over the period 1990–2013 were included in the final analysis (see Additional files 1 and 2).

### General outbreak characteristics

**Figure 2:**
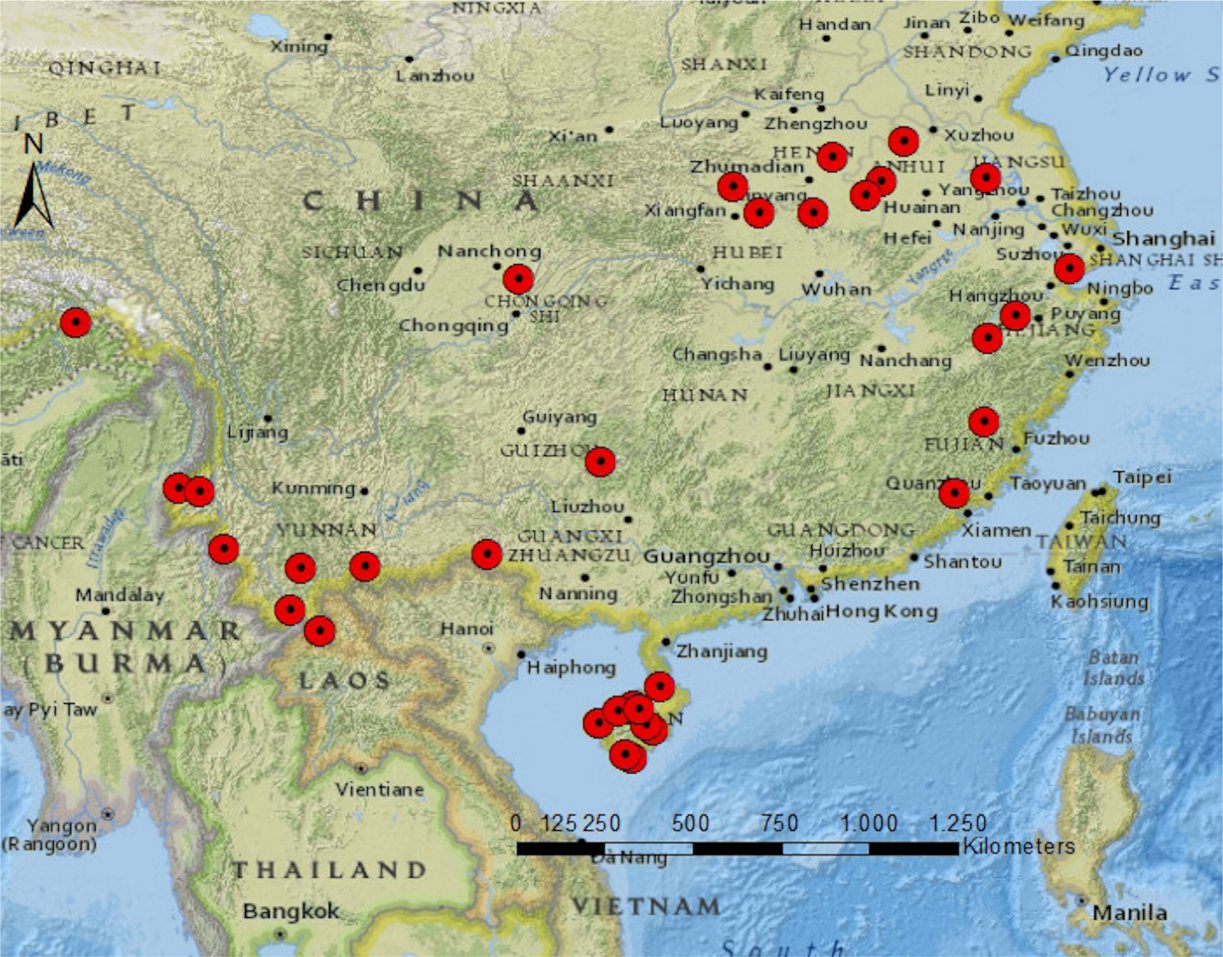
Malaria outbreak locations in China (1990–2013).

**Figure 3:**
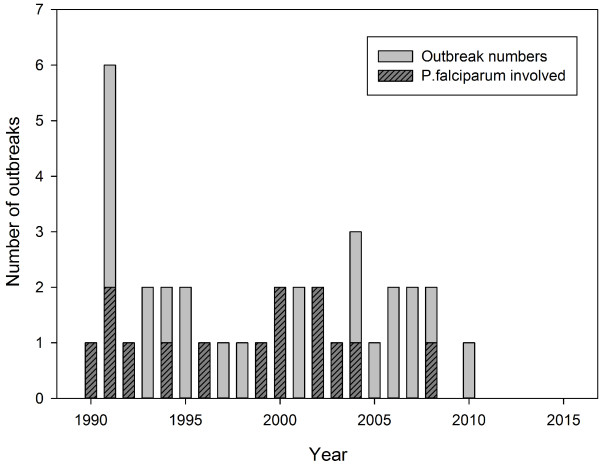
Reported malaria outbreaks in China (1990–2013).

Outbreaks were concentrated in southern China, mainly in Yunnan (n = 8) and Hainan (n = 10) Province, and in the central east (Figure 2). Outbreaks occurred rather homogenous over the study period, but from 2009 onwards, there was a sharp decline with only one further outbreak being reported (Figure 3). This last outbreak was reported from Motuo County located in Tibet Autonomous Region. The malaria species involved into outbreaks were reported in 34/36 articles; 2/34 (6%) were caused by *P. falciparum*, 20/34 (59%) by *P. vivax*, and 12/34 (35%) by both *P. falciparum* and *P. vivax* (Figure 3). Since 2005 there was only one outbreak caused by *P. falciparum,* which was in Yunnan Province (Additional files 1: Table S1).

### Outbreak causes

The causes of identified outbreaks fell into one of the four general categories, which were not mutually exclusive. Population migration was a main cause in 21/36 (58%), environmental factors in 7/36 (19%), changes of vector and host-related factors in 5/36 (14%), and operational problems of the health services in 15/36 (42%) of outbreaks (Additional file 2: Table S2). Of 36 reports, 34 were based on active field investigations (2/36 were based on retrospective data analysis). Of these, 19/34 (56%) implicated population migration, 7/34 (21%) environmental factors, 5/34 (15%) changes of vector and host-related factors, and 15/34 (44%) operational problems of the health services. Seven of 36 (19%) outbreaks were reported by more than one publication and thus assessed for information consistency. There were no differences in the main outbreak characteristics reported.

### Population migration

There are two subcategories: (1) population movement from an endemic to a non- or low-endemic area (15/21, 71%); and, (2) population movement from a non- or low-endemic area to an endemic area (7/21, 33%). The main reasons for outbreaks caused by the first subcategory were Chinese workers having migrated to an endemic area (province or country) where they became infected and introduced the malaria parasite to their home on return (7/15), Chinese guest workers from endemic areas (3/15), Chinese tourists returning from an endemic country (1/15), and non-specified migration causes (4/15). One example is the return of a local villager from a working trip to Myanmar in 2008, which was followed by a *P. vivax* outbreak in a village in Yunnan Province [17]. The main reason for the second subcategory is population resettlement (2/7) and individual labour movement (5/7). For example in another village of Yunnan Province, an outbreak of 39 malaria cases occurred in 2008, with 31/39 affected migrants from a high-latitude area [18].

### Environmental factors

There are three subcategories: (1) climatic change (1/7, 14%); (2) man-made breeding sites (3/7, 43%); and, (3) natural disasters (3/7, 43%). The one outbreak attributed to climate factors was caused by heavy rains. Due to improved breeding opportunities of the main vectors, *Anopheles sinensis* and *Anopheles minius*, in a large village in Yunnan Province (28,787 population), which had reported only 27 cases from May to September 1991, 849 malaria cases occurred in October 1991 (596/849 *P. falciparum*) [19]. This was the largest outbreak in Yuanjiang River valley since 1949 [20]. With regard to man-made mosquito breeding sites, the three outbreaks reported were mainly caused by irrigation and deforestation projects. A typical outbreak related to irrigation was documented in a village in Hubei Province, where only 19 malaria cases occurred from 1996 to 2000, but 578 malaria cases were reported in 2001 [21]. The popular conversion of dry areas into wetland for large-scale lotus plantations also contributed to increasing malaria susceptibility. For example, Shangshui County in Henan Province had reported less than five cases annually from 1989–1999, but due to malaria importation in connection with lotus farming, 1,777 malaria cases were reported in 2001 [22,23].

In the year 2005, a large fire disaster in a village in Guizhou Province led to much decreased livestock (e g, cattle) and widespread poverty, which supported increased malaria transmission (as presented also in the discussion). The village had very low annual malaria incidence rates (3 ~ 4/10,000) before 2005, but after the fire, in 2006, there were 46 malaria cases (611/10,000) [24]. Other examples of disaster-associated outbreaks are floods which have increased mosquito breeding sites in Anhui province [25].

### Vector and host-related factors

There are two subcategories: (1) increased human-vector contact (3/5, 60%); and (2) increased vector capacity (2/5, 40%). An example of the first subcategory is the reported outbreak in a school of Qiongzhong County, Hainan Province, in 1994. During this event, 72 *P. vivax* cases occurred in staff members and students because the use of bed nets had been stopped. Before this outbreak, there has been no malaria in this area for three consecutive years [26]. Another example exists from one city located in Henan Province. In this area, there were no malaria cases reported during the period 1992–2002; the first cases were seen again in 2003, and in 2006 a large outbreak was reported with 2,889 cases [27]. This outbreak was mainly caused by a combination of livestock reduction and human behaviour change [28]. An example for the second subcategory is an outbreak in a village in Henan Province in 1996, which was in part attributed to the new occurrence of the highly effective vector *Anopheles anthropophagus*[29].

### Operational problems of the health services

There are three subcategories: (1) ceased vector control measures (3/15, 20%); (2) poor case management (10/15, 67%); and, (3) weakening of surveillance (4/15, 27%).

Ceased vector control was attributed to either relaxed vigilance after a large decrease in the malaria burden or to insufficient funding support. One example is a village located in Yunnan Province, where malaria was successfully rolled back due to insecticide spraying. However, only two years after the termination of vector control measures, malaria cases increased again [30]. Problems with case management included insufficient diagnostic and treatment procedures, and stock-out of drugs. The latest outbreak recorded occurred in a small village in Tibet Autonomous Region in 2010 and was caused by stock-out of drugs [31]. Another example is a *P. vivax* malaria outbreak in a county located in Guizhou Province with 43 cases from June to July 2006, followed by a successive outbreak with 102 cases again from June to July in 2007. While the 2006 outbreak was attributed to a fire disaster (as presented above), the 2007 outbreak was attributed to non-radical treatment of *P. vivax* malaria in the previous year due to a minority population with high prevalence of G6PD deficiency [24].

An example on how weakening of surveillance can play a key role in causing malaria outbreaks was reported from a village in Yunnan Province in the year 2003. The outbreak started at the end of September with a few cases, and the first malaria deaths occurred in October. However, the responsible health authorities did not realize this outbreak until November and 60 cases (63% *P. falciparum*) had already occurred [32]. Another example is from Henan Province in 2006, which has been malaria-free from 1992–2002. A general weakening of vigilance of the authorities and the underestimate of the vector capacity of *An. sinensis* aggravated the malaria situation since the first cases were reported in 2003; there were 2,889 cases reported in 2006 [33].

## Discussion

This is the first systematic review to characterize recent malaria outbreaks in China, based on both Chinese and English publications. The study has analysed the main causes of 36 malaria outbreaks reported since 1990 in China. Malaria outbreaks occurred frequently during this time period in about half of all Chinese provinces, but there was no outbreak in the past five years. This supports the promising development of malaria burden reduction in China in recent years [3,4]. Moreover, a clear shift towards a predominance of *P. vivax* malaria was observed, which supports the assumption of *P. vivax* elimination being more difficult than *P. falciparum* elimination [34]. Population migration was the most frequently identified outbreak cause, followed by operational problems of the health services, environmental factors and changes of vector and host-related factors.

Population migration clearly is of major importance for malaria outbreaks [13]. The causal pathway can either be that populations move from an endemic to a non- or low-endemic area, leading to new or increased malaria transmission, or from a non- or low-endemic area to an endemic area, increasing the pool of susceptible individuals [35]*.* The findings from this study show that both pathways play an important role in China. Interestingly and in contrast to the situation in the USA and in European countries, where citizens with migration background often import malaria after having visited friends or relatives in their country of origin [36,37], malaria outbreaks associated with population movements in China were mainly caused by returning export labourers from endemic provinces. These are usually low social class labourers often working under conditions at increased risk of malaria (e g, mining, construction sites, forest work), who are not well protected at night due to poor accommodation, have a low education level and thus lack awareness of malaria risks, have no specific immunity, and often have limited access to health services [38]. Malaria associated with population movements is thus an important threat for elimination, but also for trying to ‘hold the line’ after having already successfully eliminated malaria [12,39]. This needs to be addressed by national programmes, e g, through active screening of returning workers especially in areas at high risk of outbreaks [12]. Early diagnosis and effective treatment would not only avoid unnecessary deaths but also secondary infections.

Operational problems of the health services were identified as another important cause of outbreaks. Here, aspects related to malaria case management were most frequently mentioned, and this concerns mainly *P. vivax*. Chloroquine in combination with primaquine has been used for more than 60 years as radical treatment for vivax malaria and is shown to still be effective in central China [40]. However, compliance and safety aspects with prolonged treatment regimens for *P. vivax* malaria remain a major challenge [41]. Minority populations in China have a high prevalence of G6PD deficiency and are thus at increased risk for haemolysis associated with primaquine treatment [42]. Innovative interventions are thus needed to address this challenge in China if the country wants to achieve the goal of malaria elimination by 2020.

Many formerly endemic areas successfully reduced the malaria burden or even achieved malaria elimination, which was sometimes followed by loosening of vigilance and consequently surveillance by the responsible health services [43]. However, in China surveillance systems have been largely strengthened in recent years and especially after the SARS epidemic [44]. For example, a web-based reporting system of infectious diseases has been implemented since the year 2003 [45]. In order to achieve malaria elimination, a robust surveillance system has been established in China that combines passive and active case detection methods with rapid response measures, including radical treatment and targeted vector control. Moreover and since 2010, China has implemented a ‵1-3-7′ surveillance strategy: one day to report a case, three days to confirm and classify the case, and seven days to conduct a local response and prevent any onward transmission [46]. Since that time, no malaria outbreaks have occurred in China.

Environmental changes such as irrigation or dam projects were also associated with malaria outbreaks in China [47]. However, whether such projects will cause malaria outbreaks depends on various factors. Malaria epidemics are less likely to derive from irrigation projects in high-transmission areas where inhabitants have natural immunity [48]; in contrast, in unstable transmission areas there is high potential for epidemics [49]. In China, most malaria-endemic areas belong to low-transmission areas. Thus, it is important to strengthen intersectoral cooperation and to establish appropriate health services, including early warning systems together with development projects [50]. Extended rainy seasons and natural disasters such as flooding and earthquakes can also contribute to malaria outbreaks [51]. A particularly interesting outbreak, which is described in this review, was attributed to a major fire disaster, where the reduction of cattle that functioned as a biological barrier largely increased human vector contact [24]. Therefore, outbreak awareness and prevention needs to accompany early responses to such disasters.

The considerable re-emergence of malaria at the beginning of 21st Century, which was mainly observed in central China, was associated with changes in vector capacity of *An. sinensis* for *P. vivax*[28,52]. In contrast to *An. anthropophagus*, which has been the main vector in this area in the past, *An. sinensis* is considered of low epidemiological significance as it usually shows exophilic and non-anthropophilic behaviour [53,54]. Malaria endemicity was sustained in areas where *An. sinensis* remained the single vector, which has been explained by a significant increase in the transmission capacity of *An. sinensis* for *P. vivax*[55,56]. These observations point to the importance of entomological surveys in areas at risk for malaria outbreaks.

This review has some limitations. As these data are from published malaria reports only, it is possible that some outbreaks not documented in the literature have been missed. Another limitation is the fact that clear reasons for outbreaks were frequently not reported in existing publications, and these were consequently not included in the analysis. The findings may also be subject to reporting bias, as report authors may be influenced by their research interests or perhaps by their affiliations (e g, with regional control programmes).

## Conclusion

The findings of this review point to the importance of sustaining quality surveillance for population movements associated with malaria transmission for preventing malaria outbreaks in China. Moreover, further strengthening of environmental and entomological surveillance as well as early case management (e g, radical treatment of vivax cases) will pave the way to achieving the final goal of malaria elimination in China.

## Competing interests

The authors declare that they have no competing interests.

## Authors’ contributions

The study has been designed by GL and OM. All authors conceived and planned the work that led to the manuscript or played an important role in the acquisition, analysis and interpretation of the data or both. All authors wrote the paper and/or made substantive suggestions for revision. All authors approved the final submitted version.

## Supplementary Material

Additional file 1: Table S1 Summary of data on malaria outbreaks in China (1990–2013) from systematic review. Click here for file

Additional file 2: Table S2 Main causes of malaria outbreaks in China (1990–2013). Click here for file
