# Supplementary material for: Malaria outbreaks in China (1990–2013): a systematic review
Source: Malar J. 2014 Jul 10;13:269. doi: 10.1186/1475-2875-13-269 (PMC4105761; doi:10.1186/1475-2875-13-269)
Supplement: Additional file 1: Table S1 — Summary of data on malaria outbreaks in China (1990–2013) from systematic review. [file 1475-2875-13-269-S1.pdf]

Additional file 1. Summary of data on malaria outbreaks in China [1990-2013] from systematic review

Place	Population involved	Start	End	Main vector	Outbreak malaria type	Definition of outbreak used	Outbreak situation	Method of data collected※
**Type-1 County†**								
Jiabutuo Reclamation area, Mengla County, Yunnan Province [1]	507	1990-01	1990-08	An.sinensis An.anthropophagus An.maculatus	P.vivax P.falciparum	WHO outbreak definition	Prevalence rate 74.8%	A
XinXing Miao Village, Qiongzhong County, Hainan Province [2]	404	1991-08	1991-08	An.dirus An.anthropophagus	P.vivax P.falciparum	WHO outbreak definition	22 cases in August	A
Mining site in Dongfang City Hainan Province [3]	31	1993-07	1993-07	An.dirus An.minimus	P.vivax	WHO outbreak definition	31 cases	A
NongKengYaWan technical School, Qiongzhong County, Hainan Province [4]	1727	1994-09	1994-11	An.minimus An.dirus	P.vivax	WHO outbreak definition	72 cases	A
Luhuitou, Sanya City, Hainan Province [5]	1720	1995-04	1995-12	An.minimus	P.vivax	WHO outbreak definition	Incidence rate 15.2%	B
Mountain area, Bianjiang Twonship, Dongfang City, Hainan Province [6]	6000	2000-06	2000-07	An.minimus An.dirus	P.vivax P.falciparum	WHO outbreak definition	52 cases	A
Rubber farming area, Guang lei harbor, Mengla County Yunnan Province [7]	781	2000-07	2000-10	An.minimus An.sinensis	P.vivax P.falciparum	WHO outbreak definition	145 cases with incidence rate 18.57%	A
Yangdang Village, Zaoyang City, Hubei Province [8]	24,092	2001-01	2001-08	An.sinensis	P.vivax	WHO outbreak definition	578 cases with incidence rate 2.4%	A
Lumiao Village, Sudian Twonship, Yingjiang County Yunnan Province [9]	243	2003-09	2003-11	An.minimus	P.vivax P.falciparum	WHO outbreak definition	30 cases and 1 death	A
JiafangXin Village, Tianya Twonship, Sanya City, Hainan Province [10]	34	2004-05	2004-05	An.minimus	P.falciparum	WHO outbreak definition	5 cases and 2 death	A
Judong Village, Xiajiang Twon, CongJiang County, Guizhou Province [11, 12]	753	2006-07	2006-08	An.sinensis An.anthropophagus An.minimus	P. vivax	Chinese guidelines	46 cases with incidence rate 6.11%	A
Villages, Yongcheng County Henan Province [13-15]	1.2 million	2006-08	2006-12	An.sinensis	P.vivax	Chinese guidelines	Incidence rate 222.7/100,000 in 2006 with 2889 cases	A
Kouyin Village, Pengying								

Twonship, Dengzhou City, Henan Province[16]	2721	2007-06	2007-09	An.sinensis	P. vivax	Chinese guidelines	34 cases	A
JuDong Village, Xiajiang Twon, Congjiang County Guizhou Province [12]	753	2007-07	2007-08	An.sinensis An.anthropophagus An.minimus	P. vivax	Chinese guidelines	102 cases with incidence rate 12.93%	A
Kangfu Village, Mengding Town, Gengma County, Yunnan Province [17]	328	2008-06	2008-07	An.vagus An.anthropophagus An.minimus	P.vivax P.falciparum	Chinese guidelines	39 cases	A
Zhu Village, Motuo County Tibet Autonomous Region [18, 19]	186	2010-06	2010-08	An.maculatus	P.vivax	Chinese guidelines	21 cases	A
**Type-2 County**								
Mingzhuang Village, Funan County, An'hui Province [20]	17366	1991-01	1991-09	An.sinensis	P.vivax	WHO outbreak definition	Incidence 13.34%	A
Tanghuayuan Village, Funan County, An'hui Province [20]	466	1991-01	1991-09	An. Sinensis	P.vivax	WHO outbreak definition	Incidence 31.55%	A
Chuanshi Twonship, Guang'an County, Sichuan Province [21]	1361	1991-08	1991-09	An.minius	/‡	WHO outbreak definition	61 cases	A
Niujiaozhai Village, Yuanyang County, Yunnan Province [22]	28787	1991-10	1991-10	An.sinensis An.minius	P.vivax P.falciparum	WHO outbreak definition	849 cases and 6 death	A
Napo County, Guangxi Province [23]	1109	1992	1992	An.minimus An.jeyporiensis Candidiensis	P.vivax P.falciparum	WHO outbreak definition	105 cases and 1 death [parasite positive rate 12.93/10,000]	A
Mining stone factory, Changliu Town, Haikou City, Hainan Province [24]	3600	1993-07	1993-09	An.minimus	P.vivax	WHO outbreak definition	153 cases	A
Four villages, Jinghong City, Yunnan Province [25]	1027	1994	1994	/	P.vivax P.falciparum	WHO outbreak definition	544 cases in 1994	A
Nadayala, Zhanzhou City, Hainan Province [5]	53	1996-08	1996-10	An.mininus	P.falciparum	WHO outbreak definition	Incidence rate 35.8%	B
Xuyi County, Jiangsu Province [26]	69126	1997-06	1997-09	An.anthropophagus An.sinensis	P.vivax	WHO outbreak definition	643 cases	A
Er'shilihe Village, Pingqiao District,Xingyang City, Henan Province [27-31]	678	1998	1999	An.anthropophagus An.sinensis	P.vivax	WHO outbreak definition	Incidence rate 20% in 1999	A

Villages, Shangshui County Henan Province [32, 33]	50,000	2001	2003	An.sinensis	P.vivax	WHO outbreak definition	1777 cases in 2001	A
Furongtian farming construction site, Hainan Province [34]	286	2002-06	2002-06	/	P.vivax P.falciparum	WHO outbreak definition	52 cases	A
Fanda Viallge, Lanyang County, Zhanzhou city, Hainan Province [34]	389	2002-08	2002-08	/	P.vivax P.falciparum	WHO outbreak definition	44 cases	A
Three villagers, Jiande City, ZheJiang Province [35-38]	42700	2004-08	2004-10	Culex An.sinensis	P. vivax	WHO outbreak definition	11 cases	A
Pingdi Village, Tengchong County,YunnanProvince[39]	54	2008-05	2008-05	An.anthropophagus An.kunmingensis	P.vivax	Chinese guidelines	6 cases	A
**Type-3 County**								
Kejian Village, Jian'ou County, Fujian Province [40,41]	2561	1991-09	1991-10	An.sinensis	P. vivax	WHO outbreak definition	24 cases	A
Liufang Village, Longjuan Twonship, An'xi County, Fujian Province [42, 43]	380	1995	1995	An. sinensis	P. vivax	WHO outbreak definition	38 cases	A
Pu'er County, Yunnan Province [44]	184839	1999	1999	An. sinensis An. maculatus	P.vivax P.falciparum	WHO outbreak definition	Incidence rate 3.89/10,000 in 1999	A
Two villages, Qinshi Town Changshan County, Zhejiang Province [45]	3678	2004-10	2004-10	/	P. vivax	Chinese guidelines	5 cases	A
Honghe Town, Jiaxing City, ZheJiang Province [46]	10000	2005	2005	An. sinensis	/	Chinese guidelines	4 cases	A

※A= Active outbreak investigation [quantitative and qualitative]; B= Based on retrospective data analysis;

†based on malaria country reports of the years 2006-2008 from Action Plan of China Malaria Elimination [2010-2020]. Type I: local infections detected in three consecutive years and annual incidences ≥1/10,000; Type II: local infections detected in three consecutive years and at least in one year the annual incidence < 1/10,000 and >0 ; Type III: no local infections reported in the last three years;

‡No information available
