# Supplementary material for: Malaria outbreaks in China (1990–2013): a systematic review
Source: Malar J. 2014 Jul 10;13:269. doi: 10.1186/1475-2875-13-269 (PMC4105761; doi:10.1186/1475-2875-13-269)
Supplement: Additional file 2: Table S2 — Main causes of malaria outbreaks in China (1990–2013). [file 1475-2875-13-269-S2.pdf]

Additional file 2. Main causes of malaria outbreaks in China [1990-2013]

Place	Outbreak main causes									
	Population migration		Environmental factor			Vector and host related factors		Operational problems of health service		
	From endemic to low/non-endemic area	From low/non-endemic to endemic area	Climatic changes	Man-made breeding sites	Nature disasters	Increased human vector contact	Increased vector capacity	Vector control ceased	Poor Case management	Weakening of surveillance
**Type-1 County †**										
Jiabutuo Reclamation area, Mengla County, Yunnan Province [1]		Yes						Yes		
Xinxingmiao Village, Qiongzhong County, Hainan Province [2]	Yes								Yes	
Mining site in Dongfang City Hainan Province [3]		Yes								
Nongkenyawan technical school, Qiongzhong County, Hainan Province [4]						Yes				
Luhuitou, Sanya City, Hainan Province [5]	Yes									
Mountain area, Bianjiang Twonship, Dongfang City, Hainan Province [6]		Yes							Yes	
Rubber farming area, Guang lei harbor, Mengla County Yunnan Province [7]		Yes								
Yangdang Village, Zaoyang City, Hubei Province [8]				Yes						
Lumiao Village, Sudian Twonship, Yingjiang County Yunnan Province [9]	Yes									Yes
Jiafangxin Village, Tianya Twonship, Sanya City, Hainan Province [10]									Yes	

Judong Village,Xiajiang Town Congjiang County, Guizhou Province [11, 12] [2006]					Yes					
Villages, Yongcheng County Henan Province [13-15]							Yes			Yes
Kouyin Village, Pengying Twonship, Dengzhou City, HeNan Province[16]									Yes	
Judong Village, Xiajiang Twon, Congjiang County Guizhou Province [12][2007]									Yes	
Kangfu Village, Mengding Town, Gengma County, Yunnan Province [17]		Yes								
Zhu Village, Motuo County Tibet Autonomous Region [18, 19]						Yes			Yes	
**Type-2 County**										
Mingzhuang Village, Funan County,An'hui Province [20]					Yes					
Tanghuayuan Village, Funan County, An'hui Province [20]					Yes					
Chuanshi Twonship, Guang'an County, Sichuan Province [21]	Yes									
Niujiaozhai Village, Yuanyang County, Yunnan Province [22]			Yes					Yes		
Napo County, Guangxi Province [23]										Yes
Mining stone factory, Changliu Town, Haikou City, Hainan Province [24]	Yes									
Four villages, Jinghong City, Yunnan Province [25]		Yes		Yes						
Nadayala, Zhanzhou City, Hainan Province [5]	Yes									

Xuyi County, Jiangsu Province [26]									Yes	Yes
Er'shilhe Village, Pingqiao District,Xingyang City, Henan Province [27-31]							Yes	Yes	Yes	
Villages, Shangshui County Henan Province [32, 33]	Yes			Yes					Yes	
Furongtian Farming construction site, Hainan Province [34]	Yes									
Fanda Viallge, Lanyang County, Zhanzhou city, Hainan Province [34]									Yes	
Three villagers, Jiande City, Zhejiang Province [35-38]	Yes									
Pingdi Village, Tengchong County, Yunnan Province [39]	Yes									
**Type-3 County**										
Kejian Village, Jian'ou County Fujian Province [40, 41]	Yes					Yes				
Liufang Village, Longjuan Twonship, An'xi County, Fujian Province [42, 43]	Yes									
Pu'er County, Yunnan Province [44]	Yes	Yes								
Two villages, Qinshi Town Changshan County, Zhejiang Province [45]	Yes									
Honghe Town, Jiaxing City, Zhejiang Province [46]	Yes									

†based on malaria country reports of the years 2006-2008 from Action Plan of China Malaria Elimination [2010-2020]. Type I: local infections detected in three consecutive years and annual incidences ⩾1/10,000; Type II: local infections detected in three consecutive years and at least in one year the annual incidence < 1/10,000 and >0 ; Type III: no local infections reported in the last three years;

Bibliography

1. Che LG, Li XL, Yang YM, Li CF, Zhang YL: **A survey on malaria outbreak in Jiabutuo Reclamation Area,YunNan Province [in Chinese].** *Chinese Journal of Parasitic Disease Control* 1992, **5:**81-83.

2. He YJ, Huang RJ, Ji WQ, Liu S: **Investigation of a vivax malaria outbreak in XinXiangMiao Village [in Chinese].** *HaiNan Medical Journal* 1993, **4:**3-4.
3. Wang JZ: **A vivax malaria outbreak in a mine field in HaiNan Province [in Chinese].** *People's Military Surgeon* 1995:11-12.
4. Duan JS, Zhang YB, Liang HJ, Chen GY, Zhu MF: **Control of a vivax malaria outbreak in QiongZhong County [in Chinese].** *Chinese Journal of Parasitology and Parasitic Diseases* 1999, **17:**270.
5. M.H. L, Wu KC, Chen WJ: **A review and analysis of focus outbreak of malaria in areas with Anopheles mimus as vector in Hainan Island.** *China Tropical Medicine* 2009, **9:**805-806.
6. Zhu QX: **Analysis of a sporadic outbreak in mobile population in mountainous area of HaiNan Province [in Chinese].** *China Tropical Medicine* 2002, **2:**239-241.
7. Sun XD, Huang GZ, Zhang ZX, Liu H: **Investigation of malaria outbreak in rubber farming development zone in GuangLei Harbor,Mengla County [in Chinese].** *Journal of Practical Parasitic Diseases* 2011, **9:**111-112.
8. Zuo SL, Chen GY, Huang GQ, Gui AF, Pei SJ, HU LQ, Chen CY, Yang DL, zhang WJ, Y.H. Y: **Analysis of the Factors Causing Malaria Local Outbreak in Anopheles Sinensis Area,Zaoyang City,Hubei Province [in Chinese].** *Gonggong Weisheng Yu Yufang Yixue* 2002, **13:**6-7.
9. Yang J, Fang XL, Lin YX, Zou J: **Survey and management of a sporadic outbreak of malaria in village on the Myanmar-China border [in Chinese].** *China Tropical Medicine* 2005, **5:**477.
10. Chen JF, LV CQ, Luo PZ, Luo QM: **Survey and treatment of a sporadic outbreak of falciparum malaria in a low endemic areas of Sanya City [in Chinese].** *China Tropical Medicine* 2004, **4:**978-979.
11. Sheng HF, Zhen X, Shi WQ, Xu JJ, Jiang WK, Wang DQ, Tang LH: **Factors Affecting Malaria Outbreak in Congjiang County of Guizhou Province.** *Chinese Journal of Parasitic Disease Control* 2007, **25:**225-229.
12. N.B. W, Long QQ, Luo T, Wang SH, Zhou J, Tang DL, Deng MM, li SP: **Analysis of the outbreak reasons of malaria for 2 years in JuDong Village of CongJiang County in GuiZhou [in Chinese].** *Modern Preventive Medicine* 2008, **35:**3629-3631.
13. Zhang HW, Su YP, Zhou GC, Liu Y, Cui J, Wang ZQ: **Re-emerging malaria in YongCheng city of HeNan Province [in Chinese].** *Chin J Vector Bio & Control* 2007, **18:**42-44.
14. Zhou GC, Zhang HW, Su YP, Zhou SS: **Epidemiologic Analysis of Malaria Outbreak in Yongcheng County of Henan Province in 2006 [in Chinese].** *Journal of tropical medicine* 2008, **8:**381-383.
15. Pan JY, Zhou SS, Zheng X, Huang F, Wang DQ, Shen YZ, Su YP, Zhou GC, Liu F, Jiang JJ: **Vector capacity of Anopheles sinensis in malaria outbreak areas of central China.** *Parasites & vectors* 2012, **5:**136.
16. Liu ZL: **Evaluation of management of malaria outbreak in DengZhou City of HeNan Province [in Chinese].** *Chinese And Foreign Medical Research* 2012, **10:**80-81.
17. Pu LQ, Hong WX, Zeng KT: **Investigation on one endogenous malaria outbreak situation around the border region in the Yunnan Province [in Chinese].** *Journal of Pathogen Biology* 2009, **4:**479-480.
18. Zhang ZX, Zhou HN, Zhao XT, Chang FX, Wang HJ, LI XJ, Zhuoma YJ, Ciren QZ, Bianma ZM, Sangdan LM, et al: **Epidemiological Survey on Malaria Situation in Motuo County of Tibet China.** *Chinese Journal of Parasitic Disease Control* 2008, **26:**343-348.
19. ZhuoMa YJ, Wang HJ, CiRen WM, Wu S, Xu GJ, Zhang R, Hu SL, Xu HM: **Investigation of a malaria outbreak in Motuo County, Tibet Autonomous Region [in Chinese].** *Int J Med Parasit dis* 2011, **38:**270-272.
20. Yang YY: **An investigation of local Plasmodium vivax malaria outbreak [in Chinese].** *Anhui Journal of Preventive Medicine* 1997, **3:**71.
21. Zhang BR, Chen ZS, Li YF, Zhou XY, Chen YZ, Meng GR, Yang SQ, Zhang CB, Li XC, Tang RH: **An Investigation of imported malaria outbreak [in Chinese].** *Chinese Journal of Parasitology & Parasitic Diseases* 1995, **13:**12.
22. Dai Q, Guo HP, Ou GR, Yang XB: **A report of falciparum malaria outbreak in a Mid-Levels District [in Chinese].** *Disease Surveillance* 1994, **9:**15-16.
23. Lin YJ: **An Investigatio of two malaria outbreaks in NaPo County [in Chinese].** *GuangXi Preventitive Medicine* 1997, **3:**220-221.

24. Si YZ, Pang XJ, Cai HL, Lin CF, Liang ZT, Kong XQ, Su AF, Fu JC, Chen SD: **An Analysis of a local malaria outbreak in Mining factory in Changliu of HaiKou City [in Chinese].** *HaiNan Medical Journal* 1995, **6:**1-2.
25. Che LG, Yang CJ, Zhang YL, Li XL: **Investigation of malaria outbreaks in JingHong City [in Chinese].** *Journal of Practical Parasitic Diseases* 1996, **4:**86.
26. Zhao LY, Gao Q, Fan JH, Yuan SG: **An Investigation of local malaria outbreak in XuYi County [in Chinese].** *Chinese Journal of Schistosomiasis Control* 1999, **11:**56-57.
27. Shang LY, Chen JS, Liu H, A.M. L, Su YJ, Gu ZC, Luo MZ, ZHEN X: **Investigation on anthropohilic index of anopheles in the outbreak site of malaria.** *Chinese Journal of Parasitic Disease Control* 2000, **13:**257.
28. Shang LY, Chen JS, Gu ZC, Zhen X, Liu H, Su YJ, A.M. L, Luo MZ, Qian HL, Tang LH: **Investigation on malaria outbreak in area of the disease basically eliminated in HeNan Province [in Chinese].** *Chinese Journal of Parasitic Disease Control* 2001, **14:**81-83.
29. Yang ZY, Wu XT, Bai Q, Dai XJ: **Surveillance on malaria in Pingqiao District of Xinyang City [in Chinese].** *Chinese Journal of Parasitic Disease Control* 2003, **16:**361.
30. Xu BL, Su YP, Shang LY, Zhang HW: **Malaria control in Henan Province, People's Republic of China.** *The American journal of tropical medicine and hygiene* 2006, **74:**564-567.
31. Sleigh AC, Liu XL, Jackson S, Li P, Shang LY: **Resurgence of vivax malaria in Henan Province, China.** *Bulletin of the World Health Organization* 1998, **76:**265-270.
32. Chen JS, Li P, Shang LY, LI XH, Zhao JH: **Investigation of epidemiological factors in a malaria outbreak in Shangshui County, HeNan Province [in Chinese].** *HeNan Journal of Preventitive Medicine* 2004, **15:**153-155.
33. Hao JL, Wu WM, Dou CY: **Investigation and control of malaria outbreaks in 2001-2003 in Shangshui county [in Chinese].** *Chinese journal of parasitology & parasitic diseases* 2004, **22:**333.
34. Yang JL, Lin JS, Chen GZ: **A report of management of two malaria outbreaks [in Chinese].** *Chinese Journal of Epidemiology* 2003, **24:**927.
35. Jin XY, Xu WM, Shi SF, Zhu SJ, Wang H, Yang Y, Xu XP, Zhu TP: **Control and investigation of a local malaria outbreak [in Chinese].** *Chinese Journal of Health Laboratory Technology* 2007, **17:**1507-1508.
36. Xu XP, Zhu TP, Wang WQ: **Investigation of malaria outbreak in SanDu Township, Jiande City [in Chinese].** *ZheJiang Preventive Medicine* 2007, **19:**34.
37. Xu XP, Wang WQ, Zhu TP: **Analysis of malaria epidemic situation and trend in Jiande from 2004 to 2007.** *Journal of Pathogen Biology* 2008, **3:**2-3.
38. Zhu TP, X.P S, Wang WQ, Wang J: **Epidemiologic analysis of epidemic situation in two malaria outbreak spots.** *Chinese Rural Health Service Administration* 2009, **29:**843-844.
39. Duan YZ, Li SG, Kang XH, Yin SQ, Sun XD: **A point-like outbreak caused by secondary transmission from an imported malaria vivax case [in Chinese].** *Int J Med Parasit Dis* 2013, **40:**57-59.
40. He RB, Wu HF, Wu JY, Xu CS: **Epidemiological factors and prevention strategy of vivax malaria outbreak in pre-elimination stage [in Chinese].** *Chinese Journal of Parasitic Disease Control* 1996, **14:**158-159.
41. Xu BH, Zhen ZJ, Liu QS, He XH, Wu JJ, Huang BF, Wu JY, He RB, Li LS, Deng YQ: **Investigation on epidemiological factors in vivax malaria outbreak area with An. Sinensis [in Chinese].** *Chinese Journal of Parasitic Disease Control* 1994, **7:**297-298.
42. Wang ZS, Li GQ, Cai BG: **Investigation of a local malaria outbreak in Anxi County of FuJian Province** *Chinese Journal of Parasitology and Parasitic Diseases* 1998, **16:**156.
43. Liao YH: **Malaria epidemiology overview of AnXi County, FuJian Province between 1950-1997 [in Chinese].** *Chinese Journal of Parasitic Disease Control* 2000, **18:**32.
44. Zhou DC: **Malaria outbreak in 3 coffee plantation farm in Puer County [in Chinese].** *Journal of Practical Parasitic Disease* 2001, **9:**144.
45. Xu QH, Dai JE, Shou SL: **Investigation on an imported vivax malaria outbreak.** *ZheJiang Preventive Medicine* 2006, **18:**26.
46. Yao LN, Xu X, XIa SR, Chen HL, Yang TT, Ruan W, Yao SR, Yu KG: **Evaluation on the effect of ongoing surveillance of malaria outbreak focus.** *ZheJiang Preventive Medicine* 2011, **23:**19-22.
